# Insights into Reproductive Immunology and Placental Pathology

**DOI:** 10.3390/ijms252212135

**Published:** 2024-11-12

**Authors:** Dariusz Szukiewicz

**Affiliations:** Department of Biophysics, Physiology & Pathophysiology, Faculty of Health Sciences, Medical University of Warsaw, 02-004 Warsaw, Poland; dariusz.szukiewicz@wum.edu.pl

The formation of a daughter organism as a result of the fusion of an egg and a sperm cell, followed by the implantation of the embryo, the formation of the placenta, and the further growth of the embryo and then fetus until delivery, poses particular challenges for the immune system [1,2]. The inextricable link between reproduction and immunity is related to the mother’s body becoming immunotolerant, which allows the semiallogeneic fetus to grow properly in the uterus. It has been shown that this is not a state of generalized immunosuppression but rather a selective adaptation of maternal immune recognition and tolerance in relation to paternal class I human leukocyte antigen C (HLA-C) molecules and nonclassical human leukocyte antigen A Ib (HLA-Ib) molecules on placental trophoblast cells of fetal origin [3,4,5,6]. With the decidua, the placenta forms the maternal–fetal interface, enabling interactions between immune cells and decidual stromal cells and trophoblast cells, a key mediator in this maternal–fetal immune interaction [7]. In this way, it is possible to transport maternal antibodies selectively to the fetus to fight infection while ensuring immunotolerance, preventing the fetus from being attacked by the maternal immune system [8,9]. Evidence that immune status profoundly influences reproductive health can be found in recent studies indicating that up to 20% of unexplained infertility in women and men may be attributable to immune dysfunction [10,11]. It is, therefore, not surprising that the issue of “immune infertility” in relation to both women and men has been the starting point for a rapidly increasing number of basic and clinical studies in recent years [12,13,14,15,16,17].

Under a more precise title, this Special Issue is a continuation of three Special Issues from the “Reproductive Immunology and Pregnancy” series [18,19,20], which confirmed the dynamic and comprehensive development of reproductive immunology and immunology during pregnancy, including placental immunopathology. Five original articles and three extensive review papers were published (their numbers are marked in bold when cited in this Editorial).

Immune responses are closely regulated by a vast array of costimulatory and coinhibitory pathways called “immune checkpoints” [21]. Incorporated into the immune system, these pathways modulate the duration and intensity of immune responses and maintain self-tolerance by preventing certain stages of acquired immunocompetence during T-cell-mediated immunity [22]. The expression of immune checkpoints and their related miRNAs in immune cells is necessary to maintain immune system homeostasis within the maternal–fetal interface [23,24,25,26]. Recently, it has been shown that immune checkpoints exist not only as cell membrane-bound systems of receptors and ligands but also in soluble forms (soluble immune checkpoints) [27]. These molecules, whose complex interactions with antigen-presenting cells (APCs), T lymphocytes, and trophoblast cells are the subject of intensive research, include sGalectin-9, TIM-3, sLAG-3, sCD80, sCD86, sVISTA, sNectin-2, and sCD155. Preliminary research has shown that all the mentioned soluble immune checkpoints could serve as biological markers of a healthy, physiological pregnancy [28]. Moreover, unlike during normal pregnancy, in the serum of pregnant women with recurrent spontaneous abortion, statistically significant increases in sLAG-3, sCD80, and sCD86, as well as reduced concentrations of sGalectin-9, sTIM-3, and sCD155, were detected. Therefore, further research is recommended to determine whether soluble immune checkpoints and their ligands may be used as biomarkers in the diagnosis of recurrent pregnancy loss [28].

Also known as silent miscarriage, missed abortion is a special type of spontaneous abortion in which the dead embryo or fetus is retained in the uterus for a period of time (days or weeks), during which the usual symptoms of miscarriage may not occur. Missed abortion is a very common complication that occurs in approximately 15% of all clinically recognized pregnancies [29,30,31]. Despite the identification of many potential etiological factors associated with missed abortion (e.g., genetic, hormonal, uterine anatomical, metabolic, infectious, environmental, and immunological factors), in the vast majority of cases, it is not possible to determine the specific cause of pregnancy loss [32,33]. Although the importance of immunologic dysregulation as a cause of “autoimmune miscarriage” has been demonstrated in the etiology of missed abortion, the exact molecular mechanisms are still poorly understood [7,34].

Human leukocyte antigen G (HLA-G) is a nonclassical HLA class I molecule composed of four membrane-bound (HLA-G1, -G2, -G3, and -G4) and three soluble (HLA-G5, -G6, and -G7) isoforms [35]. This histocompatibility antigen, which is also an immune checkpoint/soluble immune checkpoint molecule, has distinct immunomodulatory, anti-inflammatory, and tolerogenic properties that are crucial to the protection of the fetus against destruction by the mother’s immune system, ensuring maternal–fetal tolerance [36,37,38]. In another effort to understand the role of the membrane and soluble HLA-G molecules in the context of the immune pathomechanism of missed abortion, maternal–fetal interface tissue and serum samples, respectively, were examined [39]. Healthy pregnant women experiencing missed abortion were compared with healthy women in early pregnancy who were directed toward elective pregnancy termination. However, no significant differences were noted between the groups in terms of the spatial expression of HLA-G at the maternal–fetal interface or the soluble HLA-G concentration in their serum. Moreover, in women with missed abortion and women with normal early pregnancy, comparable numbers of T and gamma-delta (γδT) lymphocytes were found in the peripheral blood and decidua. Interestingly, missed abortion was not associated with altered extravillous trophoblast invasion into the uterine blood vessels or the increased cytotoxicity of γδT cells. In conclusion, the authors stated that the assessment of HLA-G production within the maternal–fetal interface and the HLA-G concentration in the serum cannot be used as markers for normal pregnancy or missed abortion. Further research is warranted because these results have not been confirmed by the latest research by other independent researchers [40].

Both miscarriage, including missed abortion, and stillbirth refer to a pregnancy with a pathological course, resulting in its premature end. However, a miscarriage is usually defined as the loss of a fetus before the 20th week of pregnancy, whereas stillbirth is frequently defined as fetal death after 20 weeks (according to other definitions: >24 to >28 weeks), with a minimum birthweight of 350 g (approximately 20 weeks) [41]. Globally, in 2019, an estimated 2 million babies (90% uncertainty interval [UI] 1.9–2.2) were stillborn at 28 weeks or more of gestation, whereas the global stillbirth rate was 13.9 stillbirth (90% UI 13.5–15.4) per 1000 total births [42]. Although the main causes and risk factors for stillbirth are known (e.g., intrapartum complications, hypertension, diabetes, infection, congenital and genetic abnormalities, placental dysfunction, and pregnancy continuing beyond 40 weeks), in up to 50% of stillbirth cases, the cause remains unexplained [43,44,45]. As syncytiotrophoblasts, which are formed by the fusion of mononuclear cytotrophoblasts, are responsible for nutrient and gas exchange in the human placenta, studies on the expression of syncytiotrophoblasts markers in placentas from women who experienced idiopathic stillbirth represent another attempt to understand the pathomechanism of fetal death [46,47,48]. The rationale for such studies is that throughout pregnancy, continuous changes in gene expression pathways occur in the placenta, facilitating the regulation of fetal growth, maintaining immunological tolerance, and modulating metabolic processes according to the needs of the fetus [49,50]. Therefore, the analysis of the dynamics of changes in the placental transcriptome creates promising prospects for understanding the functional state of this particular organ in a given period of pregnancy [51]. For example, in placentas from women who experienced an idiopathic stillbirth, several genes that are syncytiotrophoblast markers were found to be downregulated. Significant downregulation of genes from the pregnancy-specific glycoprotein and chorionic somatomammotropin hormone (CSH) families as well as the placental alkaline phosphatase, kisspeptin (KISS1), and corticotropin-releasing hormone (CRH) families was detected [48]. These results may indicate that the importance of syncytial layer defects in the etiopathogenesis of idiopathic SB has been underestimated thus far. Translating these results to clinical obstetrics practice is another key challenge, as it requires extremely accurate methods of monitoring placental function and the ability to regulate the expression of specific genes [52,53,54].

Gestational diabetes mellitus (GDM) is the most common metabolic disorder during pregnancy, and its prevalence is constantly increasing with rising obesity rates [55]. GDM exacerbates oxidative stress and weakens the antioxidant state. Like other types of diabetes, it initiates a proinflammatory background that promotes diabetic complications due to increased insulin resistance [56]. One such complication, the pathomechanism of which is not sufficiently understood, is macrosomia, which means that the fetus is “large for gestational age”. It is believed that the main cause of macrosomia in individuals with diabetes is intermittent maternal and, in turn, fetal hyperglycemia, causing the increased release of insulin combined with other anabolic hormones (e.g., insulin-like growth factors and growth hormone) in the fetus [57,58]. Abnormalities in maternal lipid transport within the maternal-fetal interface, which affects the levels of free fatty acids that are available to the fetus, may be important factors contributing to macrosomia, the essence of which involves increased fetal fat deposition [59,60]. This is because inflammation and lipid metabolism are two deeply interconnected and reciprocally regulated major physiological processes [61,62,63].

The properties of the placental barrier indicate that triglycerides (TGs) contained in maternal blood lipoproteins (chylomicrons, VLDL, and LDL), unless they are endocytosed as intact lipoprotein particles, must be hydrolyzed to free fatty acids (FFAs) [64,65]. After the action of enzymes located in the microvillous membrane of the syncytium, mainly lipoprotein lipase (LPL) and endothelial lipase, cleaved FFAs are bound in trophoblast cells by cytosolic and membrane facilitative transporters, such as fatty acid translocase (CD36), fatty acid transport proteins, and fatty acid binding proteins, and then released into the placental capillaries and fetal circulation. Therefore, it was hypothesized that changes in the activity of enzymes and FFA transporters in the diabetic placenta may contribute to excessive fetal fat accretion and, therefore, macrosomia [64,66,67]. The general pattern of FFA transport within the maternal–fetal interface and the main factors that may lead to its disorders are presented in Figure 1.

In a study on the expression of proteins that are responsible for placental lipid transport, placental tissue samples from patients with full-term pregnancies complicated by GDM with well-controlled glycemia, pre-existing well-controlled type 1 diabetes (PGDM), and no diabetes (controls) were comparatively assessed [68]. Morphometric studies investigating differences in the density of vascularization between samples revealed a significant increase in the expression of fatty acid translocase (CD36) and fatty acid binding proteins 1, 4 and 5 as well as a decrease in the expression of endothelial lipase and fatty acid transport protein 4 in patients with PGDM-complicated pregnancies compared with patients with GDMG1 (GDM treated with diet only) and those without diabetes. Therefore, well-controlled PGDM does not preclude the occurrence of disturbances in the expression of lipid transporters. Nonetheless, only LPL and fatty acid transport protein 4 were found to be significant predictors of fetal birth weight [68].

Although metabolic disorders in mothers during pregnancy that are complicated by diabetes are clearly responsible for fetal macrosomia, altered macronutrient metabolism does not completely explain its pathomechanism. There is no definitive evidence from clinical practice that modifying the lifestyle (mainly changing the macronutrient composition of the diet) of a pregnant woman to achieve optimal control of glycemia and body weight, the first-line treatment for GDM, will ensure a reduction in the incidence of either GDM or macrosomia [69,70,71]. Most likely induced by impaired glucose tolerance and low-grade inflammation, the search for other factors is necessary.

Adenomyosis is characterized by the abnormal finding of endometrial epithelial cells and stromal fibroblasts in the myometrium instead of the uterine lining [72]. It differs from endometriosis, in which endometrial-type tissue grows outside the uterus [73]. The presence of islands of endometrial tissue within the myometrium elicits the hyperplasia and hypertrophy of surrounding smooth muscle cells [72,74]. Adenomyosis is a common disorder of the uterus that is associated with an enlarged uterus, heavy menstrual bleeding (HMB), pelvic pain, and infertility [75]. Owing to the insufficient understanding of the pathomechanism of adenomyosis, many theories have been proposed concerning the causes of the development of this disease [72]. The detection of epigenetic alterations in adenomyosis directs research efforts, among other foci, toward exposure to environmental pollutants, including endocrine-disrupting chemicals (EDCs), the effects of which may result in hyperestrogenism and progesterone resistance. This hormonal imbalance predisposes patients to cell proliferation, cell migration, epithelial-to-mesenchymal transition (EMT), and the invasion of endometrial cellular components into the myometrial compartment [76,77]. Animal models have made major contributions to the understanding of the roles of estrogen-mimetic EDCs in promoting the development of adenomyosis. A study in mice demonstrated that chronic exposure to nonsteroidal anti-inflammatory drugs (NSAIDs: ibuprofen and diclofenac) and 17β-ethinylestradiol (EE2) mixtures at environmental doses intergenerationally affects uterine physiology and homeostasis, particularly in the endometrium [78]. Histological analysis revealed aberrant proliferation and apoptosis, vacuolization of epithelial cells, and an increased incidence of abnormal glands in the luminal and glandular epithelium in each studied litter (F1 and F2). This model can also be used to study the pathophysiology of human adenomyosis to a certain extent. The results of this study constitute another contribution to recommendations to the relevant authorities regarding intensive actions aimed at reducing the content of EDCs (in this case, EE2 and NSAIDs) in drinking water [79,80].

Hypertension is the most common medical disorder during pregnancy, complicating 5% to 10% of all pregnancies [81]. Hypertensive disorders of pregnancy, such as gestational hypertension (GH) and preeclampsia (PE), are among the leading causes of maternal and fetal morbidity and mortality worldwide and pose a potential risk to the health of mothers and infants [82]. The increasing incidence of hypertensive disorders of pregnancy in developed countries in recent years is also disturbing [83]. Whereas the pathophysiology of GDP is multifactorial, immunological disorders that violate the necessary level of tolerance in the mother–fetal system, manifested by inflammation and changes in the activity profiles of immune cells, have become crucial in light of recent research [84]. Deficiencies in activities related to suppressor cells may be of key importance. Although many different subpopulations of effector cells can suppress each other, particular attention is given to regulatory T cells (Tregs), which express the transcription factor forkhead box P3 (FOXP3) and are extremely important for maintaining immune homeostasis and preventing graft (fetus) versus host (mother) disease [85]. Tregs constitute a unique subpopulation of T-helper cells that secrete inhibitory cytokines, including interleukin 10 (IL-10), transforming growth factor-beta (TGF-β), and interleukin 35 (IL-35) [86]. Through IL-10 and membrane-bound TGF-β dependent pathways, Tregs can inhibit CD8+ T-cell function and significantly reduce the ability of dendritic cells (DCs) to present antigens [87,88]. Recently, published detailed reviews have shown that the development of targeted therapies that facilitate the effective use of the suppressor properties of Tregs may effectively restore pregnancy immune homeostasis, thereby counteracting hypertensive disorders of pregnancy [89,90].

Regardless of its involvement in the function of Tregs and participation in the modulation of the adaptive immune system immune tolerance, comprehensive research on the role of TGF-β in normal and complicated pregnancies continues. This multifunctional cytokine shows strong and variable expression in the cytoplasm of villous syncytiotrophoblast (STB) and extravillous trophoblast cells during gestation [91,92]. Wen et al. [93] summarized the current state of knowledge on the contributions of TGF-β to implantation and placentation and embryonic development, including the nervous, respiratory, and cardiovascular systems. The results of some studies have highlighted the importance of TGF-β dysfunction in recurrent miscarriage, PE, and GDM [94,95,96,97,98,99]. Furthermore, a decreased serum TGF-β concentration increases the risk of preterm birth [100], and increased TGF-β3 levels have been found in association with gestational trophoblastic disease [101,102].

Epigenetics is defined as potentially heritable changes in gene expression that, unlike mutations, are not attributable to alterations in the DNA sequence [103]. Epigenetics contributes to the regulation of the development and physiology of the placenta [104]. Disturbed placental epigenetics caused by a wide variety of external environmental factors that act at different times and intensities may play an important role in cases involving fetal growth restriction and newborns who are small for gestational age (SGA) and may also be involved in the pathogenesis of both PE and gestational trophoblastic disease [105,106]. Among epigenetic mechanisms, such as DNA methylation or the presence of noncoding RNAs, the degree of histone acetylation is a critical epigenetic modification that increases access (expression) to a given gene or causes its silencing through changes in chromatin architecture [107,108].

Sirtuins are a group of highly conserved enzyme proteins belonging to the nicotinamide adenine dinucleotide (NAD+) family of histone deacetylases that are critically involved in the functioning of the body at the cellular level, contributing to numerous biochemical processes [109]. In addition to regulating the degree of histone acetylation, sirtuins are involved in the regulation of the cell cycle and energy metabolism as well as the processes of cell differentiation, growth, and apoptosis [110]. Importantly, sirtuins participate in the cellular response to various types of environmental stressors, mechanical injury, pathogens, toxic compounds in the environment, and naturally occurring agents that damage cells, such as ultraviolet light and nutrient or oxygen deprivation, among others. The intensity of action of most of the abovementioned stressors in the human placenta is particularly high [111]. For this reason, a detailed understanding of the significance of changes in sirtuin expression in the human placenta may provide valuable information regarding placental physiology and the pathomechanisms of placental dysfunction [112].

Among the seven sirtuins known in humans, much of the latest placental research has focused on sirtuin-1 (silent information regulator 2 homolog 1 or SIRT1), which is particularly associated with the inflammatory response, autophagy and the cell response to oxidative stress [113,114,115]. SIRT1 is linked to placental development by controlling trophoblast cell invasion and remodeling spiral arteries. SIRT1 is expressed by the STB throughout normal gestation. Placental SIRT1 expression is decreased in the placenta accreta spectrum, which is defined as the attachment of the placenta to the uterine wall, to varying degrees [116]. Moreover, SIRT1 regulates senescence in syncytialized trophoblasts and is significantly downregulated in cases of premature placental aging in PE as well as fetal growth restriction [117,118]. Therefore, restoring normal SIRT1 expression may be an ambitious therapeutic challenge in cases of selected pregnancy pathologies. For example, multiple SIRT1-related signaling pathways with potential beneficial effects on vascular endothelial cells in PE are presented with a detailed description in Figure 2.

## Figures and Tables

**Figure 1:**
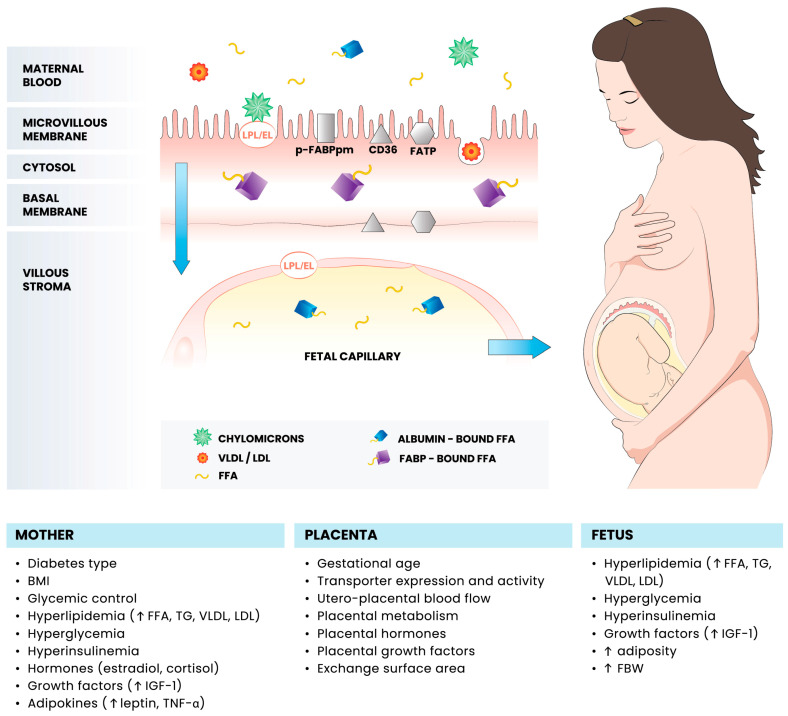
Schematic diagram of the maternal–fetal free fatty acid transfer in pregnancies complicated by diabetes mellitus. Adopted from [68]. BMI, body mass index; CD36; fatty acid translocase; EL, endothelial lipase; FABP, fatty acid binding protein; FATP, fatty acid transport protein; FBW, fetal birth weight; FFA, free fatty acids; p-FABPpm, placental plasma membrane FABP; IGF-1, insulin-like growth factor 1; LDL, low-density lipoprotein; LPL, lipoprotein lipase; TG, triglycerides; TNFα, tumor necrosis factor alpha; VLDL, very-low-density lipoprotein; up arrows indicate increase.

**Figure 2:**
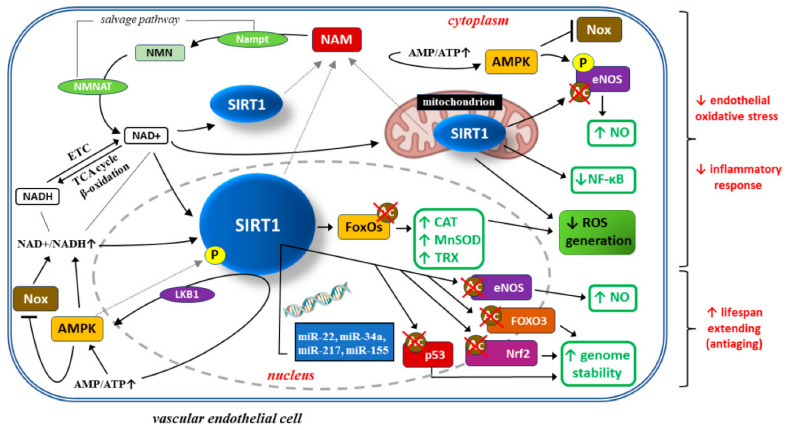
Beneficial effects of SIRT1 on vascular endothelial cells in preeclampsia (PE): limiting oxidative stress, inflammatory response, and cellular ageing. Adopted from [115]. The arrows in the boxes: up arrows indicate increase, down arrows indicate decrease. SIRT1 activity depends on the oxidized form of nicotinamide adenine dinucleotide (NAD+), which is generated from its precursor—nicotinamide mononucleotide (NMN)—by the enzyme nicotinamide-(mono)nucleotide adenylyltransferase (NMNAT) [119,120]. Although the main site of SIRT1 synthesis is the nucleus, its activity is also observed in the cytoplasm and mitochondria [119,121]. The level of NAD+ is determined by NAD+ synthesis from the salvage pathway or NAD+/reduced form (NADH) ratio. Mitochondrial redox metabolism within the electron transport chain (ETC) is crucial for SIRT1 level, because NAD+/NADH and AMP/ATP metabolism result from the tricarboxylic acid (TCA) cycle and β-oxidation or oxidative phosphorylation, respectively [122]. NAD+ is required in the SIRT1-mediated deacetylase reaction. This reaction also generates nicotinamide (NAM), which then enters the salvage pathway. Nicotinamide mononucleotide adenyltransferase (Nampt), catalyzing the conversion from NAM to NMN, is the rate-limiting enzyme in this pathway. NMN is thereby converted to NAD+ by NMNAT. The increases in NAD+/NADH ratio and AMP/ATP ratio observed during caloric restriction are well-known inducers of SIRT1. SIRT1 attenuates oxidative stress and inflammation to regulate vascular endothelial functions through several important signal mediators, such as AMP-activated protein kinase (AMPK), nicotinamide adenine dinucleotide phosphate (NADPH) oxidases (Nox), endothelial nitric oxide synthase (eNOS), and forkhead transcription factors of the O class (FOXOs) [113,123]. SIRT1 can stimulate AMPK via the modulation of upstream AMPK kinase such as liver kinase B1(LKB1), suppressing the production of reactive oxygen species (ROS) and inflammatory response in human umbilical vein endothelial cells (HUVECs), whereas AMPK influences SIRT1 deacetylation activity by increasing cellular NAD+ levels or directly phosphorylating (P) SIRT1. Increased AMP/ATP ratio induces endothelial AMPK, which in turn suppresses Nox expression and Nox-induced ROS production [124]. AMPK-dependent phosphorylation and SIRT1-dependent deacetylation of eNOS leads to an increase in local nitric oxide (NO) concentration. Moreover, SIRT1 deacetylates FoxO proteins and thus stimulates FoxO-dependent antioxidative enzymes, such as catalase (CAT), manganese superoxide dismutase (MnSOD), and thioredoxin (TRX), eliminating ROS from endothelial cells and thus preventing endothelial dysfunction [123,125,126]. SIRT1 protects endothelial cells from senescence by regulating signaling pathways dependent on tumor protein p53 (p53), eNOS, transcription factor nuclear factor erythroid 2-related factor 2 (Nrf2), and FOXO3. Expression of these proteins can, in turn, be regulated at the level of translation by several micro-RNA molecules, such as mi-R217, mi-R34a, mi-R155, and mi-R22 [127,128,129,130,131,132,133]. Optimization of NO concentration and genome stability extend the average lifespan of endothelial cells.
